# A Case of Heavy Paragonimiasis Involving a Multi-Organ Presentation with Hypereosinophilia and Hepatic Migration

**DOI:** 10.4269/ajtmh.25-0392

**Published:** 2025-12-16

**Authors:** Hidemasa Akazawa, Hideharu Hagiya, Shinnosuke Fukushima, Kenta Nakamoto, Kohei Oguni, Yohei Manabe, Mio Kokubo-Tanaka, Haruhiko Maruyama, Fumio Otsuka

**Affiliations:** ^1^ Department of Infectious Diseases, Okayama University Hospital, Okayama, Japan;; ^2^ Department of Bacteriology, Okayama University Graduate School of Medicine, Dentistry and Pharmaceutical Sciences, Okayama, Japan;; ^3^ Department of General Medicine, Okayama University Hospital, Okayama, Japan;; ^4^ Department of Pharmacy, Okayama University Hospital, Okayama, Japan;; ^5^ Division of Parasitology, Department of Infectious Diseases, Faculty of Medicine, University of Miyazaki, Miyazaki, Japan

## Abstract

Paragonimiasis is a parasitic disease caused by the genus *Paragonimus*, typically transmitted through the ingestion of raw or undercooked freshwater crabs. Although pulmonary involvement is typical, ectopic migration to subcutaneous or hepatic sites is rare and poses diagnostic challenges. A case of paragonimiasis in a 29-year-old Cambodian woman residing in Japan who presented with painless subcutaneous nodules on the chest and abdomen is reported in the current study. She had consumed raw crab 4 months before presentation. Laboratory findings revealed marked eosinophilia (21,000/*µ*L) and elevated immunoglobulin E (5,049 IU/mL). Computed tomography scans revealed pleural effusion, club-shaped pulmonary consolidation, funicular hypodense lesions in the liver, and subcutaneous lesions. Stool and sputum test results were negative for parasite eggs; however, a microplate ELISA indicated a *Paragonimus westermani* infection. The patient responded well to 3 consecutive days of praziquantel (75 mg/kg/day) and was discharged a week later. This case highlights the importance of considering paragonimiasis in patients with unexplained subcutaneous nodules and eosinophilia, especially when supported by dietary risk factors.

## INTRODUCTION

Paragonimiasis is a parasitic infection caused by lung flukes of the genus *Paragonimus*, which belong to the class Trematoda and order Plagiorchiida.^1^ These flatworms possess suckers and have a complex lifecycle that involves freshwater snails and crustaceans as intermediate hosts, as well as mammals as definitive hosts. Infection occurs through ingesting raw or undercooked freshwater crabs, crayfish, wild boar meat, or venison that harbors the infective metacercariae.^2^ In Southeast Asia, the cultural practice of eating raw freshwater crabs contributes to the disease’s prevalence.^3^

Globally, paragonimiasis affects ∼20 million people annually, with a high burden in the Asian countries, including China, Korea, Japan, Thailand, and Vietnam.^4^ Although the incidence is low in Japan, with ∼10–49 annual cases, cases in young Asian women have been increasingly reported.^3^^,^^5^ Clinical presentations involve respiratory tract symptoms, such as cough, hemoptysis, and chest pain.^5^^,^^6^ Extrapulmonary involvement, including subcutaneous nodules and hepatic migration, is an important clinical feature but is rarely observed.^5^^,^^7^

Herein, a domestic case of paragonimiasis presenting as painless subcutaneous nodules and hepatic migration after raw crab consumption in Japan is reported.

## CASE REPORT

A 29-year-old woman originally from a rural village in Cambodia presented with painless abdominal masses that had developed ∼3 months prior. Over the subsequent 1 to 2 months, additional subcutaneous nodules appeared in the chest region without systemic symptoms, such as fever or weight loss. She developed a cough and sputum production 10 days before presentation, which prompted her to seek medical attention at a local clinic. Laboratory testing revealed marked eosinophilia, with an eosinophil count of 14,000/*µ*L. Computed tomography (CT) imaging revealed left-sided pleural effusion, ascites, and enlargement of the left iliopsoas muscle. She was subsequently referred to the study hospital for further evaluation and management of her eosinophilia. She had no significant past medical history and was not taking any regular medications. Notably, her dietary history included the consumption of raw freshwater crabs caught and sold domestically in Japan 4 months earlier. She had been residing in Japan for 4 years and had not traveled abroad since immigrating.

**Figure 1.**
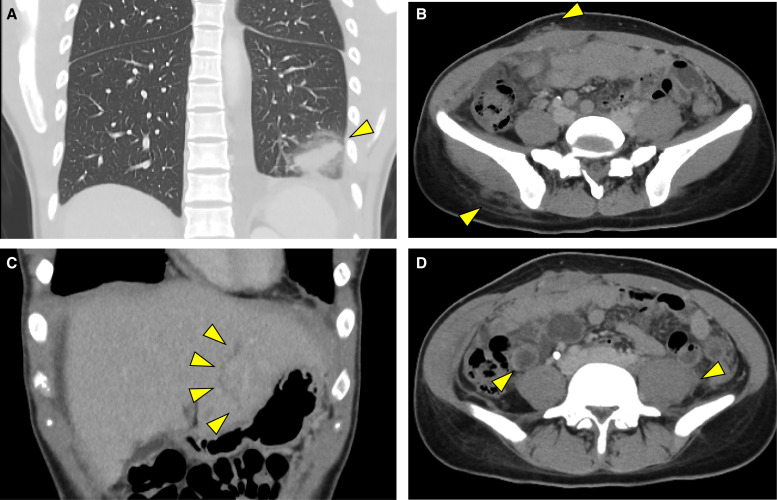
Computed tomography findings for the patient. (**A**) Club-shaped consolidations in the lower lobe of the left lung. (**B**) Subcutaneous nodular lesions in the abdominal wall and buttocks. (**C**) Funicular low-density area in the liver, indicating hepatic migration. (**D**) Enteritis and enlargement of the left iliopsoas muscle.

**Figure 2.**
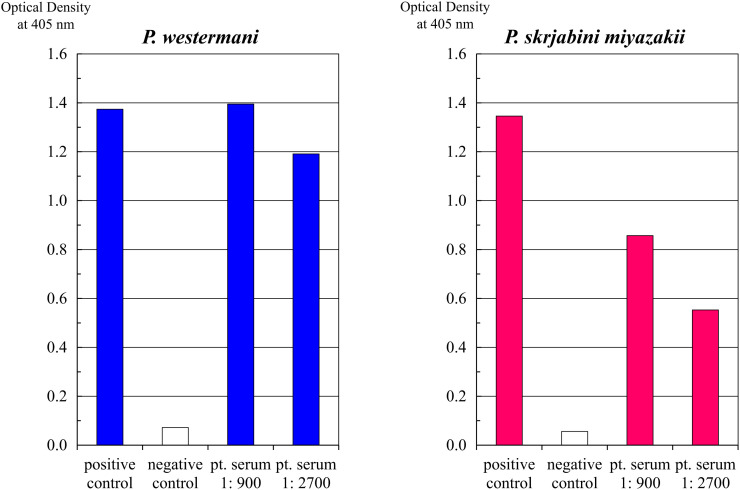
Results of microplate ELISA using *Paragonimus* antigens.

On admission, the patient’s general condition was stable, with a cough, sputum production, and mild fatigue, and her vital signs were as follows: blood pressure of 105/79 mm Hg; heart rate of 104 beats per minute; respiratory rate of 16 per minute; saturation of percutaneous oxygen at 97% on room air; and body temperature of 36.6°C. On physical examination, only painless subcutaneous masses ∼2 cm in size were palpable in the right lower abdomen and right anterior chest. No other remarkable findings, including abdominal pain, were noted. Laboratory tests revealed the following results: white blood cell count of 32,150/*µ*L; eosinophil count of 21,000/*µ*L; C-reactive protein level of 1.68 mg/dL; aspartate aminotransferase level of 49 U/L; alanine aminotransferase level of 84 U/L; alkaline phosphatase level of 210 U/L; gamma-glutamyl transpeptidase level of 38 U/L; lactate dehydrogenase level of 343 U/L; creatine kinase level of 105 U/L; and immunoglobulin E level of 5,049 IU/mL (Table 1). Contrast-enhanced chest and abdominal CT imaging revealed club-shaped consolidations in the lower lobe of the left lung and pleural effusion, subcutaneous nodular lesions in the abdominal wall and buttocks, a funicular low-density area in the liver, evidence of enteritis, and enlargement of the left iliopsoas muscle (Figure 1). Magnetic resonance imaging of the brain revealed no evidence of central nervous system involvement. A histopathological examination of the subcutaneous nodule revealed infiltration of eosinophils and neutrophils, along with necrotic tissue, but no parasitic organisms were identified. Stool and sputum examinations for parasitic eggs were performed twice each during hospitalization using direct smear microscopy of fresh feces and concentrated sputum specimens; however, no eggs were detected. Serological tests using a microplate ELISA demonstrated elevated IgG levels against somatic antigens of *Paragonimus westermani* (*P. westermani*) and *Paragonimus skrjabini miyazakii* (*P. skrjabini miyazakii*). The optical density values of the patient’s serum at a 1:900 dilution exceeded those of the positive control for *P. westermani*, whereas those for *P. skrjabini miyazakii* were lower, suggesting *P. westermani* infection (Figure 2).

**Table 1:** Laboratory data on admission

Laboratory Data	Value	Unit
Complete blood count		
WBC	32,150	/*µ*L
Neut	20.5	%
Ly	8.0	%
Eo	65.5	%
Mo	2.0	%
RBC	4.43 × 10^6^	/*µ*L
Hb	13.3	g/dL
PLT	41.6 × 10^4^	/*µ*L
Chemistry		
TP	8.4	g/dL
Alb	3.3	g/dL
AST	49	U/L
ALT	84	U/L
γ-GT	38	U/L
T-bil	0.4	mg/dL
LDH	343	U/L
CK	105	U/L
UN	11.4	mg/dL
Cr	0.79	mg/dL
CRP	1.68	mg/dL
Na	135	mEq/L
K	4.2	mEq/L
Cl	104	mEq/L
Ca	8.8	mg/dL
IgE	5,049	IU/mL

Alb = albumin; ALT = alanine aminotransferase; AST = aspartate aminotransferase; Ca = calcium; CK = creatine kinase; Cl = chloride; Cr = creatinine; CRP = C-reactive protein; Eo = eosinophil; γ-GT = γ-glutamyl transpeptidase; Hb = hemoglobin; IgE = immunoglobulin E; K = potassium; LDH = lactate dehydrogenase; Ly = lymphocyte; Mo = monocyte; Na = sodium; Neut = neutrophil; PLT = platelet; RBC = red blood cells; T-bil = total bilirubin; TP = total protein; UN = urea nitrogen; WBC = white blood cells.

On the basis of a diagnosis of paragonimiasis, the patient was treated with oral praziquantel at a dose of 75 mg/kg/day for 3 consecutive days. After treatment, the patient’s cough, sputum, and marked eosinophilia subsided, and she was discharged on admission day 7 with a clinical course. Follow-up CT imaging performed 3 months after discharge confirmed the complete resolution of all lesions.

## DISCUSSION

A case of paragonimiasis in a patient who initially presented with painless subcutaneous nodules after ingesting raw crab in Japan is described in the present study. This case highlights the importance of including paragonimiasis in the differential diagnosis of painless subcutaneous nodules, particularly in patients with eosinophilia and relevant dietary history.

*Paragonimus* species invade the human body when raw or undercooked second intermediate hosts, such as freshwater crabs, or paratenic hosts, such as wild boar, are consumed. According to a previous report, wild boar was the most common dietary source (43.2%), exceeding freshwater crab (26.4%) among Japanese patients. In contrast, among immigrant cases, freshwater crab was the predominant dietary source, accounting for 74.1%, whereas wild boar accounted for less than 1%.^6^ After penetrating the intestinal wall, *Paragonimus* larvae migrate into the peritoneal cavity, transiently move through the abdominal wall or retroperitoneum, and then re-enter the peritoneal cavity before passing through the diaphragm to reach the lungs.^3^ The most common clinical manifestations of paragonimiasis are respiratory symptoms, which typically occur 8 to 12 weeks after parasite infection. Cough (29%) and sputum (27%) are frequently observed.^6^ Delayed diagnosis or inappropriate corticosteroid administration may lead to complications such as pneumothorax.^8^ Radiologically, pleural effusion is the most common finding. Notably, pleural effusion and pneumothorax are reported more frequently in younger patients (under 50 years of age) than in older individuals.^6^ In addition, peripheral eosinophilia is observed in ∼75% of patients, serving as an important diagnostic clue for paragonimiasis.^6^^,^^9^^,^^10^ Moreover, an increase in eosinophil count, along with elevated parasite-specific serum IgM levels, is often seen in the early phase of pleuritis, whereas mild eosinophilia and elevated parasite-specific IgG are more common in the later phase.^10^ Consistent with previous reports, the present case involved a young female patient who presented with cough and sputum and was found to have pleural effusion and peripheral eosinophilia.

During the migratory stage, larvae may cause transient lesions in various organs other than the lungs. When larvae aberrantly migrate to the subcutaneous tissue, liver, or brain, ectopic paragonimiasis may manifest as subcutaneous nodules, hepatic cystic lesions, or meningitis or intracerebral mass lesions, respectively.^6^^,^^11^ Although subcutaneous involvement is frequently reported in endemic regions such as China,^12^ its prevalence in Japan is quite low, estimated at ∼4–5%.^5^^,^^6^ This difference might be partly attributable to the distribution of *Paragonimus* species or differences in infection intensity. In Japan, *P. westermani* accounts for ∼84% of cases, whereas *P. skrjabini miyazakii* comprises only ∼16%.^13^ The latter species is more commonly found in China and exhibits a greater tendency for extrapulmonary migration, potentially contributing to the higher incidence of subcutaneous involvement and atypical clinical manifestations observed in that region.^12^ In addition, high-intensity infections tend to occur more frequently in immigrants than in native Japanese populations, which may also contribute to the increased incidence of subcutaneous nodules. Hepatic involvement, which typically consists of hypodense cystic lesions,^7^ is also rare, reported in ∼5% of cases.^5^ However, the presence of a migrating, cord-like hypodense lesion in the liver, suggestive of actual “creeping,” has not been previously documented. In patients presenting with pulmonary lesions and eosinophilia accompanied by subcutaneous nodules or hepatic cord-like hypodense lesions, paragonimiasis should be considered in the differential diagnosis. A detailed dietary history, especially regarding the consumption of raw or undercooked freshwater crustaceans or wild game, should be carefully interviewed.

The diagnosis of paragonimiasis relies on both parasitological and serological tests.^3^ The direct detection of parasite eggs in stool or sputum is often limited by intermittent egg shedding, resulting in a diagnostic sensitivity of ∼12%, with only 0.2% confirmed solely by stool examination.^6^ In fact, the authors of one study reported that eggs were detected in the stool samples of only 1.9% of 365 patients with positive intradermal reactions.^14^ Moreover, sputum examinations have been shown to be more sensitive than stool examinations. Among the 40 egg-positive cases, 37 were positive for eggs in sputum, whereas only 21 were stool egg-positive.^15^ In addition, it has been demonstrated that the formol–ether concentration method yields a substantially higher detection rate of intestinal parasites (65.3%) compared with direct smear (34.7%), highlighting the limited sensitivity of direct stool smear techniques.^16^ In the present case, eggs were not detected in either stool or sputum specimens; however, because only direct smear microscopy was performed, the possibility of having missed parasite eggs cannot be excluded. The microplate ELISA is considered to provide higher diagnostic sensitivity and reliability.^3^ When paragonimiasis is clinically suspected, both sputum examination for eggs and the antibody test should be performed promptly.

In summary, the present case highlights the importance of considering paragonimiasis in patients who present with eosinophilia and pulmonary lesions when migratory subcutaneous or hepatic lesions are also observed. Although such atypical manifestations are not diagnostic on their own, they may serve as valuable clinical clues that prompt further investigation, such as an antibody test. Furthermore, heightened clinical vigilance regarding parasitic infections associated with regional dietary practices and epidemiological risk factors is essential to facilitate the prompt diagnosis and optimal therapeutic management of uncommon parasitic pathologies.
